# Beyond the Th2 paradigm: CD4+ cytotoxic T lymphocytes as key drivers of tissue damage and fibrosis in IgG4-related disease

**DOI:** 10.3389/fimmu.2026.1781462

**Published:** 2026-02-12

**Authors:** Jiayang Yi, Lanlan Jia, Tongjun Mao, Zhi Li

**Affiliations:** Department of Rheumatology, The First Affiliated Hospital of Wannan Medical College, Wuhu, Anhui, China

**Keywords:** CD4+ cytotoxic T lymphocytes, fibrosis, IgG4-related disease, immunopathogenesis, SLAMF7, tissue damage

## Abstract

IgG4-related disease (IgG4-RD) is a distinctive immune-mediated disorder characterized by multi-organ involvement, dense IgG4+ plasma cell infiltration, and storiform fibrosis. While pathogenesis has traditionally been attributed primarily to T helper type(Th) 2 cytokines (e.g., Interleukin(IL)-4/IL-10), this mechanism insufficiently accounts for the observed tissue destruction and progressive fibrosis. Emerging data highlight the extensive, oligoclonally expanded infiltration of CD4+ cytotoxic T lymphocytes (CTLs) deep within lesions. These cells possess dual cytotoxic and profibrotic properties. This review systematically elucidates the role of CD4+ CTLs as a distinct lineage and core effector population. We detail how these cells mediate the pathology linking chronic inflammation and fibrosis through direct cytotoxicity, secretion of profibrotic factors, and complex B-cell interactions. Finally, we assess the clinical potential of CD4+ CTLs as biomarkers of disease activity and as novel therapeutic targets.

## 1 Introduction

Immunoglobulin G4-Related Disease (IgG4-RD) is a recently recognized systemic fibroinflammatory disorder capable of affecting nearly every organ system (1–3). The classic histopathological triad consists of dense lymphoplasmacytic infiltration, storiform fibrosis, and obliterative phlebitis, often accompanied by significantly elevated IgG4 levels in affected tissues and/or serum (2, 4). Although glucocorticoid therapy is highly effective initially, up to 50% of patients experience relapse, and long-term disease progression frequently leads to irreversible organ fibrosis and functional impairment (5–7).

For a long time, the pathogenesis of IgG4-RD has been attributed to a T helper type(Th) 2 immune response paradigm, primarily based on the frequent allergic history reported by patients and the expression of Th2 cytokines (e.g., Interleukin(IL)-4, IL-10) within affected tissues (4, 8). However, this prevailing view faces increasingly severe challenges. Firstly, previous studies often failed to rigorously distinguish between the disease’s specific immune processes and coexisting allergic states. Secondly, a purely Th2 response poorly accounts for the significant tissue destruction, widespread collagen deposition, and defining fibrotic characteristics observed in IgG4-RD (4, 9). Consequently, there remains a critical knowledge gap concerning the core pathogenic mechanisms of IgG4-RD, necessitating the urgent identification of novel effector cells and molecular pathways.

Traditionally, CD4+ T cells are defined as helper cells, primarily responsible for coordinating the functions of other immune cells, such as B cells and CD8+ T cells. Nevertheless, a specialized subset possessing direct target cell killing capabilities—CD4+ Cytotoxic T Lymphocytes(CTLs)—is garnering attention, particularly in the contexts of chronic viral infections (e.g., Epstein-Barr virus, Cytomegalovirus, Human Immunodeficiency Virus) and tumor immunosurveillance (10). These cells highly express classical cytotoxic molecules, including granzymes and perforin, enabling them to efficiently induce target cell apoptosis in a Major Histocompatibility Complex(MHC) Class II-restricted manner (11–15). Importantly, the aberrant accumulation of CD4+ CTLs in fibrotic autoimmune diseases, such as Systemic Sclerosis and Sjögren’s Syndrome, suggests a significant profibrotic potential extending beyond their direct cytotoxic functions (4, 16–19).

Building upon this foundation, this review aims to integrate recent research findings to systematically demonstrate the mechanism by which CD4+ CTLs act as core effector cells driving the specific pathology of IgG4-RD, particularly focusing on tissue damage and fibrosis. We will thoroughly delineate their phenotypic characteristics, differentiation origins, specific pathogenic mechanisms, clinical relevance, and therapeutic implications. By attempting to transcend the conventional Th2 paradigm, we seek to provide an innovative theoretical framework for comprehensively understanding the pathogenesis of IgG4-RD and developing novel targeted therapeutic strategies.

## 2 Phenotypic landscape analysis of CD4+ cytotoxic T lymphocytes in IgG4-RD

The precise identification of CD4+ CTLs associated with IgG4-RD hinges on their unique combination of surface markers, transcription factors, and effector molecules. This characteristic molecular signature definitively distinguishes them from classical helper T subsets, including Th1, Th2, Th17, and follicular helper T cells (Tfh).

### 2.1 Core surface markers and transcriptional factors

Groundbreaking studies have designated Signaling Lymphocyte Activation Molecule Family Member 7 (SLAMF7, CD319) as a pivotal surface marker. Mattoo and colleagues were the first to identify the prominent clonal expansion of SLAMF7+ CD4+ T cells within both the peripheral blood and affected tissues of IgG4-RD patients (4). These findings were subsequently validated and expanded upon by independent cohorts (20–22), confirming SLAMF7 as a robust pathogenic signature. This observed oligoclonal expansion strongly implies an antigen-driven proliferation event originating from a limited pool of precursor cells (4, 20, 23). The expression of SLAMF7 shows a robust correlation with cytotoxicity, likely augmenting effector function via homophilic interactions that stabilize cell-to-cell contact (20, 24). Crucially, the concurrent enrichment of the chemokine receptor CX3CR1 and the terminal differentiation marker CD57 endows these SLAMF7+ CD4+ T cells with essential tissue-homing capabilities and characteristics indicative of an advanced effector state (25–28).

Regarding transcriptional regulation, CD4+ CTLs notably deviate from canonical helper subsets: they fail to express the lineage-defining master factors GATA3 (Th2) or Bcl6 (Tfh). Instead, they exhibit high expression of T-bet (TBX21) and Eomesodermin(Eomes), transcription factors intrinsically linked to cellular cytotoxicity and effector function (29, 30). Research indicates that T-bet and Eomes exert overlapping yet complementary roles in the induction of key effector molecules, including perforin, granzymes, and Interferon-γ(IFN-γ) (30, 31). Furthermore, the transcription factor Hobit is implicated in sustaining both the cytotoxic program and the tissue-resident phenotype of these cells (31, 32). This distinct and tightly regulated transcriptional network ultimately constitutes the molecular foundation driving the acquisition and long-term maintenance of the CD4+ CTLs’ lethal killing capacity and pronounced pro-inflammatory phenotype.

### 2.2 The effector molecular profile: cytotoxic granules and profibrotic cytokines

CD4+ CTLs in IgG4-RD are recognized as highly potent effector cells. They store and subsequently release classic cytotoxic granule components, including perforin, Granzyme A, and Granzyme B (4, 33, 34). Perforin functions by forming transmembrane channels on target cell membranes, which facilitates the entry of granzymes into the cytosol. Granzymes then directly initiate target cell apoptosis through the cleavage of critical substrates, such as caspases (14, 35).

Of greater pathological significance is the distinct cytokine profile secreted by these cells. Unlike the typical Th2 signature, which primarily features IL-4, IL-5, and IL-13, CD4+ CTLs predominantly generate IFN-γ, IL-1β, and TGF-β1 (4, 36–38). It is this specific molecular convergence that underpins their functional duality, equipping them to wield destructive inflammatory power while concurrently orchestrating the machinery of fibrosis. As a potent pro-inflammatory mediator, IFN-γ activates macrophages, thereby exacerbating the local inflammatory response (39). Conversely, TGF-β1 is widely recognized as one of the most powerful pro-fibrotic factors. It directly activates fibroblasts, promoting their transdifferentiation into myofibroblasts and resulting in the excessive deposition of the Extracellular Matrix (40–42). Moreover, IL-1β further amplifies inflammatory signals and acts synergistically with TGF-β to accelerate the progression of fibrosis (43, 44). Collectively, these data suggest that CD4+ CTLs function as a mobilized fibrotic driver unit, capable of precisely delivering both cellular cytotoxic and pro-fibrotic signals directly into the pathological lesion.

### 2.3 Single-cell omics evidence

Single-cell RNA sequencing (scRNA-seq) offers robust evidence for the precise identification of CD4+ CTLs within complex tissue microenvironments. Maehara et al. analyzed scRNA-seq data from affected IgG4-RD tissues (pancreas, kidney, salivary glands) (45, 46). This analysis revealed that the most pronounced clonal expansion among tissue-infiltrating T cells belonged to CD4+ T cells characterized by a cytotoxic gene signature (high expression of *Granzyme A*, *Granzyme B*, *PRF1*, *IFNG*, and *SLAMF7*). Collectively, these transcriptomic data confirm that CD4+ CTLs are the dominant and clonally expanded T cell population in IgG4-RD lesions, displacing traditional Th2 or other helper subsets.

## 3 Ontogeny and differentiation of CD4+ CTLs

The prominent oligoclonal expansion of CD4+ CTLs observed in IgG4-RD robustly indicates that their differentiation and subsequent proliferation are driven by specific antigenic stimulation (34).

### 3.1 The antigen-driven hypothesis

T-cell receptor (TCR) sequencing analyses have demonstrated that CD4+ CTLs infiltrating affected tissues possess a highly restricted TCR repertoire, where a limited number of clones achieve clonal dominance (4, 47). This phenomenon suggests that specific peptide-MHC Class II complexes may persistently activate corresponding naïve or memory CD4+ T cells, thereby driving their selective expansion and subsequent differentiation into a cytotoxic effector phenotype.

Although the specific antigens driving IgG4-RD remain unidentified, the “chronic, repetitive antigenic stimulation” model constitutes the central mechanistic hypothesis explaining the genesis of CD4+ CTLs. Potential sources for these antigens include microbial agents (such as viruses), autoantigens, and neoantigens exposed following tissue injury.

### 3.2 Potential for viral induction

Given the critical role of CD4+ CTLs in controlling latent infections such as Epstein-Barr virus and Cytomegalovirus, researchers have hypothesized that the reactivation or persistent antigenic exposure from these viruses may serve as a potential trigger for IgG4-RD (10).

Studies confirm that primary Epstein-Barr virus infection induces robust, antigen-specific CD4+ CTL responses (48, 49). In the context of chronic infection, sustained antigenic stimulation is known to propel CD4+ T cells toward a terminally differentiated cytotoxic phenotype (48, 50). However, it is crucial to acknowledge that direct evidence establishing a causal link between specific viral infections and IgG4-RD remains elusive. While the “hit-and-run” hypothesis or persistent viral antigen exposure is biologically plausible, no specific viral genome has been consistently isolated from IgG4-RD lesions across studies. Thus, the viral etiology currently serves as a theoretical framework rather than a proven mechanism.

### 3.3 Shaping the cytokine environment

A distinct cytokine milieu is indispensable for the differentiation of CD4+ T cells into the CTL phenotype. Cytokines such as IL-2, IL-12, and IL-15 strongly promote the expression of the crucial transcription factors T-bet and Eomes, concomitantly upregulating the production of key cytotoxic molecules like granzyme and perforin (51–54).

Furthermore, given that IL-21 has been demonstrated to enhance the cytotoxic function of CD8+ T cells, its potential role in the differentiation pathway of CD4+ CTLs warrants focused investigation (55, 56). Although direct evidence of IL-21 driving CD4+ CTL differentiation specifically within IgG4-RD tissues is still emerging, generally elevated IL-21 levels in IgG4-RD (typically associated with Tfh cells) could theoretically contribute to this process. In the inflammatory microenvironment characteristic of IgG4-RD, these cytokines—secreted by infiltrating plasma cells, B cells, and macrophages—likely synergize to form a unique “cytokine cocktail” that specifically drives the differentiation of naïve or central memory CD4+ T cells into CD4+ CTLs (57–59).

## 4 Core pathogenic mechanisms mediated by CD4+ cytotoxic T lymphocytes in IgG4-RD

**Figure 1:**
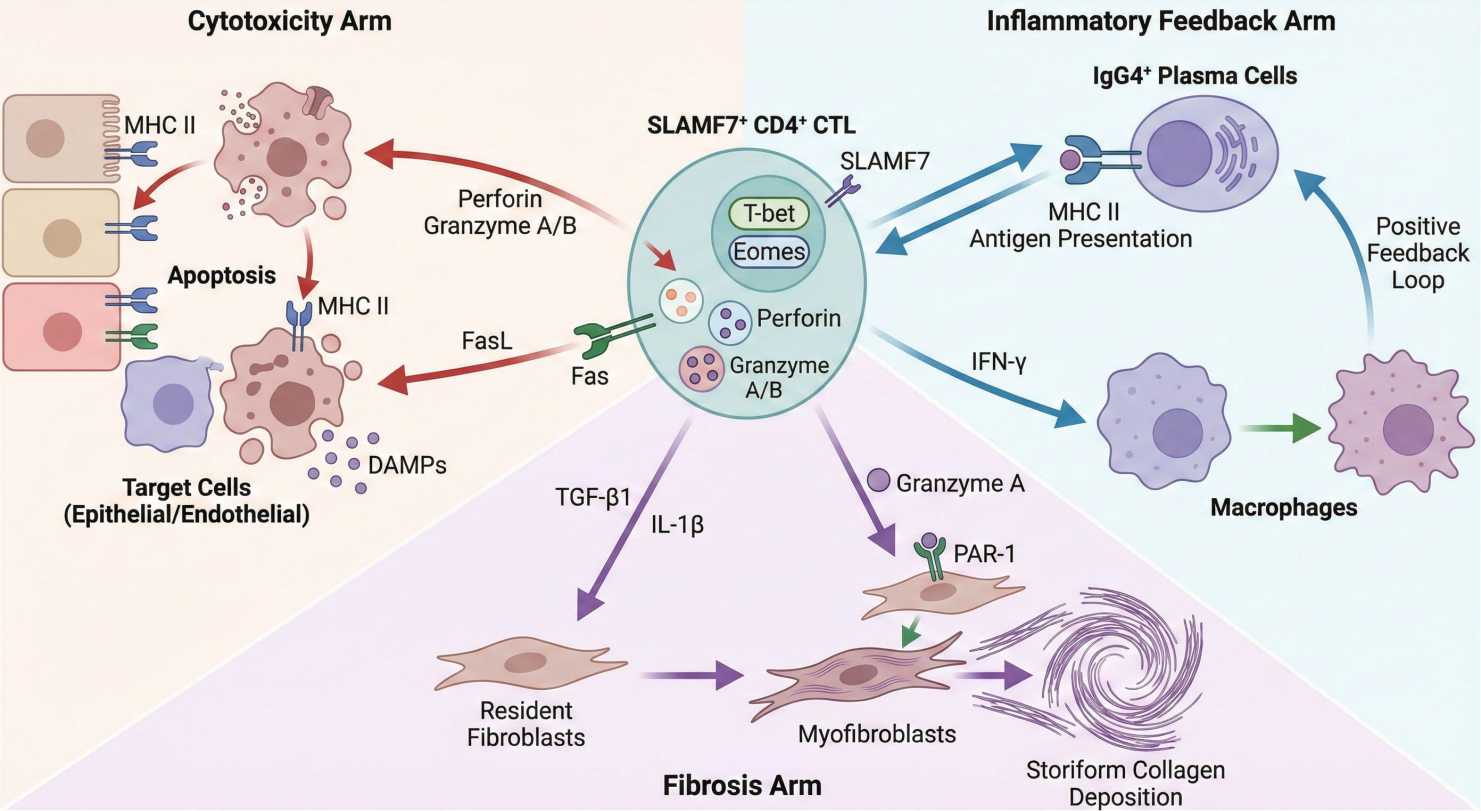
Schematic diagram illustrating the central driving role of CD4+ CTLs in the pathogenesis of IgG4-RD. Centrally positioned SLAMF7+ CD4+ CTLs drive pathological progression through three primary pathways: (1) Cytotoxic pathway (left): Induce apoptosis in MHC II-expressing epithelial/endothelial cells by releasing perforin, granzyme B, and FasL-Fas interaction, thereby releasing DAMPs to exacerbate inflammation; (2) Fibrosis pathway (bottom): Secretion of TGF-β1 and IL-1β activates fibroblasts; concurrently, extracellular granzyme A directly induces myofibroblast transformation and Dense Fibrosis by cleaving the PAR-1 receptor; (3) Inflammatory Feedback Loop (right): Interacts with IgG4+ B cells/plasma cells via MHC II for antigen presentation, forming a sustained positive feedback loop of activation, and secretes IFN-γ to activate macrophages.

CD4+ CTLs are pivotal to the pathogenesis of IgG4-RD. These cells employ multiple synergistic pathways, directly orchestrating the hallmark histopathological changes characteristic of this fibroinflammatory disorder (**Figure 1**).

### 4.1 Direct cytotoxicity and tissue injury

CD4+ CTLs primarily execute target cell lysis through two canonical pathways: the perforin-granzyme pathway and the Fas Ligand(FasL)-Fas pathway. Target cells typically express MHC Class II molecules. In affected tissues, such as salivary glands, pancreatic ducts, and bile ducts, inflammatory stimuli induce the upregulation of MHC Class II on epithelial and endothelial cells, converting these cells into key targets for CD4+ CTL attack (60–62). The release of perforin and Granzyme B directly triggers programmed cell death (apoptosis) in the target cells (35, 63). Concurrently, the interaction between FasL expressed on CD4+ CTLs and Fas on the target cell surface activates the canonical death receptor-mediated apoptotic cascade (52, 64). Of note, environmental factors such as oxidative stress can significantly enhance the susceptibility of endothelial cells to Fas-mediated apoptosis (64, 65).

This direct cellular killing is the initiating event for ensuing tissue injury and structural disruption. Apoptotic cells subsequently release abundant intracellular antigens and damage-associated molecular pattern (66), thereby amplifying the localized inflammatory cycle and providing the requisite initiating signals for subsequent tissue remodeling and fibrosis (67).

### 4.2 Critical mechanisms driving fibrosis

The induction of fibrosis stands out as one of the most critical and high-impact pathogenic contributions of CD4+ CTLs to the immunopathology of IgG4-RD. Their pro-fibrotic effects are mediated both through soluble cytokines (68) and via direct, apoptosis-independent mechanisms (69).

Cytokine-mediated fibrosis centers on TGF-β1 secreted by CD4+ CTLs, which critically drives fibroblast activation and subsequent Extracellular Matrix synthesis (40). This effect is amplified by IL-1β, which acts synergistically with TGF-β to potentiate pro-fibrotic gene expression. Crucially, these CD4+ CTLs concurrently release IFN-γ, a factor largely deemed anti-fibrotic due to its capacity to suppress TGF-β signaling and fibroblast collagen synthesis (40, 70, 71). Therefore, IgG4-RD-associated fibrosis evolves within a complex microenvironment defined by this imbalance between pro- and anti-fibrotic factors; yet, the robust pro-fibrotic signaling provided by the CD4+ CTLs ultimately prevails.

Recent studies have demonstrated that Granzyme A possesses an apoptosis-independent profibrotic function. Extracellular Granzyme A cleaves crucial Extracellular Matrix components, such as Type IV collagen and fibronectin, thereby compromising basement membrane integrity (72, 73). Crucially, Granzyme A directly activates fibroblasts by proteolytically cleaving and activating protease-activated receptor-1 (PAR-1) (74, 75). PAR-1 activation, a G protein-coupled receptor signaling event, subsequently initiates intracellular calcium mobilization, RhoA activation, and the MAPK signaling cascade. These downstream events culminate in heightened fibroblast proliferation, migration, and augmented collagen synthesis (75, 76). This mechanism effectively couples the cytotoxic effector functions of CD4+ CTLs with their capacity for tissue remodeling, allowing for a devastating “dual-mode” progression of the disease.

While CD4+ CTLs provide a robust explanation for the intensity of fibrosis via TGF-β1, IL-1β, and Granzyme A-mediated matrix remodeling, the mechanism driving the specific “storiform” (cartwheel-like) architectural pattern remains an intriguing open question. The storiform pattern likely results from complex spatial interactions between activated myofibroblasts, inflammatory cells, and the physical forces within the edematous tissue, rather than cytokine signaling alone. Whether CD4+ CTLs influence this spatial arrangement through specific cell-cell contact or localized matrix cleavage requires further high-resolution spatial transcriptomic analysis.

### 4.3 The T-B cell interaction axis

A critical and intricate interplay exists between CD4+ CTLs and the B cell/plasma cell lineage, establishing a central axis in IgG4-RD immunopathology.

While some observations note low-level expression of Bcl6 or CXCR5 on CD4+ CTLs, hinting at a potential Tfh-like function (77), the prevailing consensus classifies this population as a distinct subset. Its defining characteristic remains cytotoxicity rather than canonical T-helper activity (77, 78). Consequently, these cells may not directly dictate IgG4 class switching but instead modulate B cell activation and differentiation indirectly by orchestrating a specific inflammatory milieu.

A widely accepted model posits that activated B cells and plasma cells function as highly efficient antigen-presenting cells (APCs), sustaining the activation and clonal expansion of CD4+ CTLs through continuous antigen presentation via MHC Class II molecules (10, 50, 79, 80). This interaction establishes a potent positive feedback loop: activated CD4+ CTLs drive inflammation and subsequent tissue damage, which, in turn, releases a greater antigenic burden. B cells subsequently internalize, process, and present these liberated antigens, thereby perpetuating the stimulation of the CD4+ T cell population. Crucially, this mechanism elegantly accounts for the pronounced therapeutic efficacy of the B-cell-depleting agent Rituximab. Rituximab achieves clinical remission by not only eliminating antibody-producing cells, but also by interrupting this T-B cell axis, which leads to a concomitant decline in CD4+ CTL populations (59, 81–84).

## 5 Clinical relevance and potential biomarker utility

Beyond their clear pathological significance, the cellular frequency and functional status of CD4+ CTLs correlate strongly with various clinical parameters, establishing them as highly promising candidates for effective biomarkers. Consistent findings across numerous studies demonstrate a significant positive correlation between the frequency of CD4+ CTLs—both in the peripheral circulation and within affected tissues—and the degree of disease activity. Specifically, CD4+ CTL counts are closely associated with serum IgG4 levels, the overall burden of affected organs, and established clinical disease activity scores (4, 69).

Crucially, existing evidence further suggests a strong correlation between the degree of CD4+ CTL infiltration in lesional tissue and the severity of irreversible tissue fibrosis (69, 85). This critical association suggests that monitoring CD4+ CTLs could serve multiple clinical purposes: assessing disease activity, predicting the risk of potential relapse, and tracking the progression of organ fibrosis. Such monitoring could be achieved through detailed analysis of biopsy samples or non-invasively via flow cytometry for specific subsets, such as the SLAMF7+CD4+ T cell population. Compared to reliance solely on serum IgG4 levels, which possess inherent limitations regarding specificity and sensitivity, CD4+ CTLs offer a potentially more direct and precise metric for quantifying the intensity of the underlying pathogenic immune response.

## 6 Therapeutic implications: targeting CD4+ CTLs

A thorough comprehension of the pivotal role played by CD4+ CTLs in pathogenesis is crucial. This understanding not only aids in re-evaluating the mechanisms of action for established therapies but also illuminates promising avenues for the development of innovative treatments (**Table 1**).

**Table 1:** Overview of current and emerging therapeutic strategies targeting CD4+ CTLs in IgG4-RD.

Therapeutic strategy	Agent	Primary target	Mechanism of action on CD4+ CTLs	Development status in IgG4-RD
Direct Depletion/Inhibition	Elotuzumab	SLAMF7 (CD319)	Targets SLAMF7+ CD4+ CTLs for ADCC-mediated depletion or inhibits pathogenic homotypic interactions.	Potential/Theoretical (Approved for Multiple Myeloma)
Disruption of Differentiation Axis	Rituximab	CD20 (B cells)	Disrupts the T-B cell axis; eliminates B cells as efficient APCs, breaking the antigen presentation loop required for CD4+ CTL maintenance.	Established/Off-label Use
Blockade of Co-stimulation	Abatacept	CD80/CD86 (on APCs)	Blocks CD28-mediated co-stimulation (Signal 2), inhibiting the clonal expansion of CD4+ CTLs driven by chronic antigen stimulation.	Potential/Investigational
Inhibition of Intracellular Signaling	JAK Inhibitors (e.g., Tofacitinib)	JAK1/JAK2/JAK3	Disrupts cytokine signaling cascades (e.g., IFN-γ, IL-2) essential for CD4+ CTL activation, proliferation, and effector function.	Potential/Investigational
Broad Immunosuppression	Glucocorticoids	Glucocorticoid Receptor	Broadly suppresses T cell activation and inhibits the production of cytotoxic granules and pro-fibrotic cytokines.	Standard of Care (First-line)

### 6.1 Re-evaluation of existing therapeutic mechanisms

#### 6.1.1 Glucocorticoids

Glucocorticoids, which exert their therapeutic effects through the broad suppression of immune cell activity and cytokine production, predictably inhibit activated CD4+ CTLs.

#### 6.1.2 Rituximab

The clinical success of Rituximab is often attributed to the depletion of B cells and plasma cells. However, a more fundamental mechanism may involve the effective disruption of the critical pathogenic axis: B cell-mediated antigen presentation leading to CD4+ CTL activation. This disruption subsequently induces the reduction in CD4+ CTL counts and functional inactivation (79, 86–88). Consequently, this refined mechanistic view provides a more precise immunological rationale for the application of Rituximab in IgG4-RD.

### 6.2 Potential novel therapeutic targets

#### 6.2.1 Elotuzumab (anti-SLAMF7 monoclonal antibody)

Targeting Signaling Lymphocyte Activation Molecule Family Member 7 (SLAMF7) represents a highly forward-looking therapeutic strategy. Elotuzumab is a humanized monoclonal antibody currently approved for treating multiple myeloma. Its mechanism of action involves augmenting natural killer cell-mediated antibody-dependent cell cytotoxicity and impeding the homotypic interactions of SLAMF7 (89–93).

Crucially, SLAMF7 has been identified as a signature marker for pathogenic CD4+ CTLs in IgG4-RD, where its interactions are hypothesized to promote cellular activation and subsequent tissue infiltration (20, 24). Theoretically, therefore, Elotuzumab could achieve therapeutic efficacy in IgG4-RD, especially for refractory or relapsing cases, by either directly clearing SLAMF7-positive CD4+ CTLs or functionally suppressing them.

#### 6.2.2 Abatacept

Abatacept is a synthetic fusion protein designed to mimic Cytotoxic T Lymphocyte-Associated Antigen 4 (CTLA-4). It acts by binding competitively to CD80/CD86 molecules on antigen-presenting cells (APCs), thereby preventing the essential co-stimulatory interaction with the T cell receptor CD28 (94, 95). This mechanism effectively blocks the T cell co-stimulatory signal (Signal 2), a critical requirement for T cell activation, including that of CD4+ CTLs. Specifically targeting co-stimulatory signaling is posited as a potentially potent inhibitory strategy against the clonal expansion of CD4+ CTLs, which is often sustained by chronic antigenic stimulation.

#### 6.2.3 Janus kinase inhibitors

The activation and effector function of CD4+ CTLs are highly contingent upon cytokine-driven signal transduction. Key examples include IFN-γ signaling through the JAK1/JAK2-STAT1 pathway and IL-2 via the JAK1/JAK3-STAT5 pathway. JAK inhibitors (e.g., Tofacitinib, Baricitinib) offer a means to broadly disrupt these downstream signaling cascades, consequently suppressing the activation, proliferation, and effector functions of CD4+ CTLs (96, 97). This strategy offers a distinct potential therapeutic avenue for mitigating CD4+ CTL-mediated pathology.

## 7 Conclusion and future directions

Positioning CD4+ CTLs at the core of IgG4-RD pathogenesis offers a persuasive framework that transcends the traditional Th2 paradigm to provide a more robust explanation for the disease’s complexity. By seamlessly integrating direct tissue cytotoxicity, the release of potent pro-fibrotic factors, and pathogenic B-cell crosstalk, this unique subset precisely mirrors the hallmark pathological features of IgG4-RD characterized by the simultaneous presence of inflammatory destruction and significant fibrosis. Indeed, the role of CD4+ CTLs is now well-established, with evidence ranging from the identification of clonally expanded phenotypes and the elucidation of molecular mechanisms driving fibrosis to their correlation with clinical activity and the definition of potential therapeutic targets.

Nevertheless, further advancement necessitates resolving several critical scientific inquiries, beginning with the elucidation of antigen specificity to determine whether the driving force stems from common environmental microbes or tissue-specific autoantigens, which remains a fundamental issue in understanding etiology. Concurrently, research must define the precise differentiation trajectories using *in vivo* localization techniques to identify the microenvironmental signals instructing this phenotype. Furthermore, it is crucial to assess whether functional or phenotypic heterogeneity across organs accounts for the diverse clinical presentations of IgG4-RD. Finally, developing translational interventions requires a delicate balance between ablating pathogenic CD4+ CTLs and preserving protective immunity, a context in which clinical trials focusing on targets such as SLAMF7 represent pivotal steps toward validating this theory and ultimately delivering highly effective and personalized therapeutic regimens.
